# Perceived Infertility or Fertility Anxiety? Qualitative Insights from Young Adults Attending Reproductive Health Centers

**DOI:** 10.1177/26884844251399090

**Published:** 2025-11-19

**Authors:** Summer L. Martins, Anna K. Schulte, Christy M. Boraas

**Affiliations:** ^1^ Allina Health, Minneapolis, Minnesota, USA.; ^2^ Department of Obstetrics, Gynecology and Women’s Health, University of Minnesota Medical School, Minneapolis, Minnesota, USA.

**Keywords:** fertility, focus group, infertility, qualitative research, reproductive health, young adult

## Abstract

**Background:**

Prior studies have documented fertility concerns among adolescents and young adult women, but reasons for these perceptions have not been fully elucidated. We explored the origins and dimensions of fertility concerns in a sample of U.S. young adults assigned female sex at birth.

**Methods:**

We conducted focus groups with participants aged 18–25 years recruited from reproductive health centers in Minnesota, United States, from 2021 to 2022. Eligible participants were assigned female sex at birth, had recent penile–vaginal sex, had never been pregnant, were not attempting pregnancy, and self-identified as having ever been worried about their fertility. Transcripts were analyzed qualitatively for themes regarding: (1) reasons for fertility concerns and (2) participants’ perceptions of their current fertility.

**Results:**

Participants (*n* = 19) were predominantly cisgender and 47% were Black, Hispanic, or Multiracial. Reasons for fertility concerns emerged under three domains: personal history of unprotected sex without subsequent pregnancy, risk factors (*e.g.*, contraceptive use, environmental exposures), and psychosocial factors such as inflated perceptions of infertility prevalence. Participants who thought they would have difficulty conceiving in their current state cited mostly evidence-based risk factors like irregular menses and comorbidities; participants who thought it would be relatively easy referenced a lack of those same factors.

**Conclusions:**

Young people with fertility concerns cite myriad reasons and do not necessarily believe they are *currently* infertile. Sexual and reproductive health messaging for adolescents and young adults should broaden beyond pregnancy prevention to proactively dispel misconceptions and alleviate fears related to fertility and infertility.

## Introduction

Fertility^a^ concerns have been frequently observed in samples of U.S. adolescent and young adult women,^1–4^ even though infertility is least prevalent during these life stages.^5^ Because valid screening tools for infertility do not exist, there are no avenues for people to be completely reassured of their reproductive capacity other than attempting pregnancy. Therefore, young people may harbor unresolved fertility concerns for many years even while striving to avoid unintended pregnancy. Little research has explored how adolescents and young adults navigate this tension.

One behavior in which fertility concerns may manifest is lack of contraceptive use, as motivation for use hinges on one’s perception that they are capable of becoming pregnant. Evidence to date is mixed; some studies find that young women with fertility concerns are less likely to use contraception or to use it consistently,^1,6–8^ while others report null or conflicting findings.^4,9^ These inconsistencies could be attributable to the wide variation in how studies have measured women’s fertility concerns—as fertility fear, perceived infertility, perceived susceptibility to pregnancy, and other constructs. Notably, many measures do not explicitly differentiate women’s concerns over their *current* fertility status from anxiety about being able to achieve pregnancy in the abstract future. Qualitative research methods can help detangle these complex and interwoven constructs.

There is also a need for more empirical investigation into the drivers of fertility concerns among young people without an infertility diagnosis. In one survey, young women who perceived themselves to be infertile were asked to select possible reasons from a list, and one-third chose none of them.^4^ New insights from people of color are also needed, considering that Black and Hispanic Americans are more likely to experience fertility concerns, clinical infertility, and delays in accessing infertility treatment.^4,5,10,11^ In this qualitative study, we explore fertility concerns and perceptions in a diverse sample of young cisgender women and nonbinary individuals assigned female at birth (AFAB). Specifically, we examine both the origins of their fertility concerns and the self-appraisal of their current fertility status.

## Methods

### Setting and recruitment

We recruited participants from 18 free-standing reproductive health centers in Minnesota from August 2021 through May 2022. Study flyers invited participants to self-screen online for further eligibility assessment. Inclusion criteria were 18–25 years old, AFAB, history of penile–vaginal sex in the past 12 months, desire for future pregnancy, and affirmative response to “Have you ever worried about your ability to get pregnant?” We excluded people who indicated in the screener that they were sterile, had a clinical diagnosis of infertility, or were currently attempting pregnancy. We also excluded people who had ever been pregnant, as they have objective confirmation of their fertility status. The University of Minnesota Institutional Review Board approved the study protocol. All participants provided informed consent before completing study activities.

### Data collection

We conducted focus group sessions virtually due to considerations of the COVID-19 pandemic. The platform utilized both audio and video. Participants were encouraged, but not required, to appear on video. Our target was three to five participants per session to ensure smooth facilitation and because participants joining *via* mobile phone were limited in the number of users they could view simultaneously. Participants completed a baseline survey measuring sociodemographic characteristics and reproductive health history prior to the session and received a $20 electronic gift card at its conclusion.

The same facilitator (Black cisgender woman) led all sessions using a semi-structured focus group guide (Supplementary Data), probing participants’ knowledge, attitudes, and perceptions pertaining to fertility and infertility. She solicited participants’ connotations of “fertility” and “infertility” and then provided definitions of these terms to guide the remainder of the discussion. Two questions were particularly germane to this analysis: (1) *What are some reasons that you, or people you know, worry about their fertility?* and (2) *Now, imagine you wanted a baby right now and started trying. How easy or difficult would it be for you to get pregnant? Why?* The principal investigator also attended the sessions and met with the facilitator afterward for a recorded debrief on their observations, including similarities or differences with other sessions. We held six focus group sessions ranging from two to five participants each and lasting 23–55 minutes in duration. Five sessions were comprised of cisgender women, and one was restricted to nonbinary participants. We verified all transcripts generated by the online platform against audio recordings for accuracy and then deidentified them in preparation for analysis.

### Analysis

The study team developed a qualitative code dictionary of both deductive and inductive codes based on the focus group guide, debriefing sessions, and preliminary review of transcripts. Using NVivo 11 (Lumivero, 2015), we coded the transcripts, aggregated data for each code in queries, and exported the queries to Microsoft Word. We further synthesized the data by constructing thematic matrices summarizing emergent themes, degree of endorsement within the sample, and illustrative quotations.^12^ The principal investigator served as the primary coder and analyst, but all authors independently reviewed the raw transcripts, compiled their own reflections on the data, and contributed to drafts of analytic matrices.

We used descriptive statistics to summarize the distribution of baseline characteristics. We classified participants’ residential ZIP codes as urban or nonurban using the ZIP code approximation of Rural–Urban Commuting Area Codes.^13,14^

## Results

Of 19 focus group participants, approximately half were Black, Hispanic, or Multiracial (Table 1). Participants were predominantly cisgender female, urban, employed, and of low religiosity. Eleven (57.9%) participants were in a casual or committed relationship. Over half were using long-acting and/or hormonal methods of contraception. Use of withdrawal was also common, but most of these participants also reported using condoms or highly effective methods (data not shown).

**Table 1.** Sociodemographic and Reproductive Health Characteristics of Participants (*n* = 19)

Characteristic	*n* (%)
Race/Ethnicity	
Black, non-Hispanic	2 (10.5)
Hispanic, Latino(a), or Latinx	5 (26.3)
Multiracial, non-Hispanic	2 (10.5)
White, non-Hispanic	10 (52.6)
Gender	
Female, cisgender	17 (89.5)
Nonbinary, assigned female at birth	2 (10.5)
Age, years	
18–20	4 (21.1)
21–23	5 (26.3)
24–25	10 (52.6)
Urban residence	16 (84.2)
In school	11 (57.9)
Employed	16 (84.2)
Has household members who receive public benefits	4 (21.1)
Importance of religion in daily life	
Very important	1 (5.3)
Somewhat important	2 (10.5)
A little important	5 (26.3)
Not at all important	11 (57.9)
Sexual orientation	
Straight or heterosexual	11 (57.9)
Bisexual or fluid	7 (36.8)
Other	1 (5.3)
Relationship status	
Single	7 (36.8)
In a casual or dating relationship	3 (15.8)
In a serious or committed relationship	8 (42.1)
Unknown	1 (5.3)
Current contraceptive method(s)^a^	
Condom	6 (31.6)
Implant	1 (5.3)
Intrauterine device	6 (31.6)
Oral contraceptive pill	4 (21.1)
Withdrawal	7 (36.8)
None	3 (15.8)

^a^ Categories are not mutually exclusive.

### Reasons for fertility concerns

When elaborating on the reasons why young people worry about their fertility, participants most often shared firsthand perspectives but also commented on what they have noted among their peers or the broader community. We observed themes that emerged under three domains: History of unprotected sex, risk factors, and psychosocial factors.

#### Domain 1: History of unprotected sex

Some participants cited their personal experience of having unprotected sex, knowingly or unknowingly, and not getting pregnant afterward as a cause for their fertility concerns. One participant described her difficulty in making sense of her experience: “I was in a serious relationship and wasn’t taking care of myself [using contraception] and nothing happened. Was it the *timing*? Was it the *person*? Or was it *me*?” Others mentioned their lack of pregnancy after unprotected sex over multiple years or with multiple partners.

#### Domain 2: Perceived risk factors

Participants identified several factors that they perceived as increasing a person’s risk of infertility, thus triggering fertility concerns (see themes and quotations in Table 2). One prominent theme was contraception, with participants expressing concerns over the deleterious effects of using certain methods or using contraception for long periods of time. At times, they chose to discontinue their method in order to preserve their future fertility. In addition to sharing their personal concerns and experiences, participants also noted that contraceptive concerns were prevalent among their friends or people in general. One participant was reassured by a nurse that contraception cannot cause permanent fertility damage but remarked that “you probably can Google it and find a bunch of data that supports that [birth control use can impact future fertility].”

**Table 2.** Perceived Risk Factors for Infertility Cited by Focus Group Participants

Risk factor	Example quotations
Contraception	“I’ve got some friends who don’t want to use hormonal birth control because they’re worried that it will permanently affect their body when they do want to get pregnant.”
Longer duration of use	“I stopped taking [Depo Provera] and I have still yet to get back my monthly period. It is incredibly irregular—maybe, like, once or twice a year I’ll have it. And I think that’s just a little worrisome after being on birth control for so long.”
Hormones	“I used to have the IUD…but then I started getting a lot of strange pains and it made me very scared because I’d like to have the option to have kids and I don’t want to damage my uterus.”
Intrauterine devices	
Overuse of emergency contraception	
Family history	“My sister is going to be infertile. She takes medications that will affect her ability to reproduce and they are medications for a condition that runs in our family. So I have become concerned that it could also happen to me.”
	“I have family history of just different issues with their uterus so that also makes me a little nervous.”
Health status and behaviors	“I have a friend that just recently had a miscarriage and it scares me because she has lived a very healthy lifestyle. …[I]f somebody that’s ‘healthier’ than I am suffered a miscarriage what makes me—I’m always eating junk food or am lacking exercise, not staying hydrated as I should—be able to carry full term?”
Eating disorders	“I think about as well, like, alcohol use or drug use, more specifically marijuana use. Some people wonder if that could affect fertility.”
Irregular menses	“My cousin was having troubles getting pregnant for a long time because she had an eating disorder when she was a teenager. And I guess I’m worried about that because of mine.”
PCOS	
Physical inactivity	
Poor diet	
Substance use	
General “health issues” or “lifestyle”	
Environmental exposures	“I think there’s just so many more chemical substances in the world and our food and in everything else that we do that, in a kind of pessimist way, *one* of those things has got to be hurting my fertility so I’m probably infertile.”
General “chemicals”	“I think about medications…as well as recently with the Covid-19 vaccine, I know there are some discussions about possible impacts on fertility, so I think a lot about like general medications that birthing people may be on and like could raise concerns with fertility.”
Pharmaceuticals	“I’ve been on tons of medications for depression, anxiety, mood disorders, and one of the things that they always put in, there is ‘oh could cause reproductive health issues’ and it’s like, ‘Wwwwhat kind?!’ Because they don’t specify that. They just say *could* cause and so I’m wondering how much cause could it have.”
Unknown	“[W]ith your body, anything could happen to it at any time and you don’t know. No matter if you go to the doctor for your annual checkup, there’s always something that can end up happening that you might not be aware of.”
	“That typically is how I feel about female health where it’s like a gray area. No one [expletive] knows what’s going on.”

IUD, intrauterine device; PCOS, polycystic ovary syndrome.

The second theme centered on family history and genetics. Participants with mothers, sisters, and other female relatives who experienced fertility problems—or had medical conditions with known links to infertility—worried that these risks would extend to them. Third, participants referenced a variety of risk factors related to health status and behaviors including reproductive health disorders (*e.g.*, polycystic ovary syndrome, irregular cycles), other medical conditions, and general health and “lifestyle.” For one participant, a miscarriage experienced by a more health-conscious friend made her doubt her own ability to conceive and carry to term. The fourth category consisted of environmental and pharmaceutical exposures. A potential link between the COVID-19 vaccine and infertility was raised in more than one group, although some participants characterized this association as misinformation. Some participants spoke broadly about potential reductions in fertility due to “medications,” while others specifically named prescription drugs used to treat mental health disorders. Last, a few participants talked about the influence of unknown factors—for example, unrecognized diagnoses and limitations in medical knowledge related to women’s health.

#### Domain 3: Psychosocial factors

Themes in this category are related to values and norms surrounding fertility and childbearing. Most prominently, fertility concerns were rooted in the highly valued personal goal of having children in the future. As one participant described, “Having kids is something that I definitely see in my future so it’s just anxiety about not being able to control how easy it’ll be.” Some participants further clarified that their fertility goals included experiencing pregnancy and childbirth—for example, “It is like a *dream* of mine; being able to go through the process of pregnancy and delivering a child is something I desire deeply. And the thought of not having it is extremely scary.” Another participant described a shift in her fertility goals, from being “very, like, pro no-baby” to being open to a theoretical pregnancy, after meeting the right partner. This change prompted her to be more vigilant about protecting her fertility, and she reported worry about possible “damage” to her uterus from an intrauterine device.

A second psychosocial theme outlined a phenomenon whereby participants’ exposure to infertility narratives and statistics led to inflated perceptions of its prevalence, therefore exacerbating their fertility anxiety. Participants described greater visibility and awareness of infertility in general and more infertility-based storylines in scripted media, in particular. While this shift was often viewed as a positive one—for example, “it’s a good thing that a lot of people are talking about it these days”—participants nonetheless drew a connection to greater fertility concerns. One participant articulated this tension:

“I appreciate when people share [their infertility] because it reminds you that everyone’s going through their own struggles. Personally, though, it does make me nervous when I hear about it, because it makes it seem like it can happen to anyone. And since I don’t really know how fertile I am, it just makes me think like, ‘Hmm, this could happen to me.’”

Other participants shared their immediate reactions to stories and data related to infertility: “Well, why would it be easy for me?” and “Oh, God, what if it’s me? What if I’m next?” One participant described how an emphasis on infertility in their information environment created a negative bias: “You usually only hear about the issues and not…like, ‘Hey, this is how many successful births has happened this year!’”

Two additional themes were less prominent but notable. First, participants discussed the influence of cultural norms on their fertility concerns. One noted, “In our culture, a lot of your worth is based off of your ability to have children. …If I’m infertile, would I ever be really satisfied with my life? Would I feel fulfilled?” while another felt pressure to be “the bearer of children” in their immigrant family. Participants also felt the influence of gender norms—for example, women “are definitely conditioned to think about fertility more”—and being at a life stage where more peers are starting their families. Second, a few participants connected fertility concerns to a pessimistic mindset (“I think people have a tendency to doubt themselves when they’re trying to do something.”) or keeping expectations low—“If I set the bar low that I’m infertile, I can’t be disappointed because it’s already worst-case scenario.”

### Perceptions of current fertility

When participants were asked to appraise their current fertility status, their responses and rationales varied. (We were unable to classify three participants due to conflicting or missing responses.) The largest group (*n* = 7) thought it would be relatively easy, or take the average amount of time, to get pregnant if they started trying. They gave reasons that included a history of regular menstrual cycles, their young age, and no knowledge of factors “working against” them. These participants did not mention having comorbidities or other risk factors such as family history. As one participant said, “when I wasn’t on hormonal birth control my cycle was exactly 28 days to the T, like almost to the hour, like it was right on time. So I would say I’d be fairly confident.” Nonetheless, some still expressed worry over their fertility, citing the influence of unrecognized risk factors and “bad habits” like drinking alcohol and poor diet.

A second group (*n* = 5) occupied the opposite end of the spectrum, believing it would be difficult or harder than average for them to conceive. Their perceptions were rooted in their personal history of unprotected sex with no resulting pregnancy, irregular menses, family history of infertility, and health problems. Responses included:

“Based on my history…I’m like *strongly convinced* that I can’t get pregnant. …I’ve had a couple of partners that, like, really tried and nothing ever happened.”

“I feel like it would be difficult for me to predict because I have very irregular cycles. To predict when I am ovulating or when I’m not.”

“I would be pretty…surprised if it happened really easily just because my mom had issues with fertility. …With fibroids running in my family, I know that I have a high possibility of having those.”

The third, and smallest, group (*n* = 4) could not confidently speculate on their current fertility status. Responses included “50/50” and “honestly have no idea.” Some mentioned having comorbidities that could affect their fertility. Others added that they hoped it would not be difficult to conceive, but ultimately do not know until they attempt pregnancy.

## Discussion

This study sheds light on the phenomenon of perceived infertility among older adolescents and young adults—populations who are statistically at peak fertility.^5^ The reasons why young people worry about their reproductive capacity have not been fully characterized in the literature. In our sample of young, predominantly cisgender women seeking reproductive health care, some reasons for their fertility concerns echoed those reported in prior studies: lack of pregnancy after unprotected sex,^4,15,16^ perceived harm from contraceptive methods,^17,18^ family history,^4,19^ and personal medical history.^4,15,19^ Concerns over permanent damage from contraception, especially if used for many years, were particularly prominent. This finding underscores the importance of contraceptive counseling that dispels myths about long-term effects on fertility.

We also identified some novel contributors to fertility concerns. First, encountering information about infertility or other people’s fertility struggles did more than just foster awareness for some participants; it seeded doubts over their future fertility. This finding highlights the ways in which awareness efforts in particular (*e.g.*, infertility-based storylines in television) may backfire. Risk perceptions are informed by numerical information such as prevalence but become heightened for threats that appear frequently in the media, are viewed as beyond one’s control, and are experienced by family or close friends—all factors cited by participants in our study.^20^ Second, participants worried about pharmaceutical and environmental exposures and their impact on fertility. Prior qualitative studies have described these concerns in samples of young people^21^ but not in those who question their fertility status. Third, when identifying potential causes of infertility, participants invoked the mystery of the human body and factors that are beyond the current limits of medical knowledge. These notions are reflected in the clinical diagnosis of “unexplained infertility,” which up to 30% of couples will receive after assessments reveal no known causes for their failure to conceive a pregnancy.^22^ Last, some participants pessimistically assumed their fertility was impaired as a way of buffering against future disappointment. This distinctive and novel finding points to a potential psychological benefit of keeping fertility expectations low.

Asking participants to assess their likelihood of conceiving were they to attempt pregnancy now, rather than in the abstract future, revealed important distinctions in their fertility concerns. Some thought it would be difficult based on their having risk factors for infertility, others expected relative ease based on their lack of those factors, while the remainder said it was too hard to predict. All of these appraisals align with the scientific literature on infertility and the probabilistic nature of conception. Moreover, our findings suggest that young people with fertility concerns do not necessarily believe themselves to be infertile in their current state. Rather, they are anxious about experiencing infertility in their future when pregnancy is desired. Future studies should carefully delineate between these two constructs in their measures of fertility concerns.

Fertility concerns among young adults may be influenced by their baseline vulnerability to anxiety. National data show an almost twofold increase—from 7.7% to 14.7%—in reported anxiety among 18- to 25-year-olds from 2012 to 2018.^23^ Prevalence was highest among young adults compared with all other age groups, a pattern also observed in national surveys during the COVID-19 pandemic.^24^ Anxiety is also more common in women versus men.^23,24^ While not explored in our study, recent and tumultuous shifts in the U.S. policy landscape restricting people’s access to abortion, *in vitro* fertilization, and life-saving pregnancy care are newer sources of anxiety for young adults contemplating their reproductive futures.

Our study has several limitations. First, our sample was limited to patients seeking care in a network of reproductive health centers in a single U.S. state. Fertility perceptions in other populations, particularly those who do not access reproductive health care, are likely to differ. Our sample was also predominantly nonreligious. Religiosity is associated with both higher *intended* and *actual* fertility, which may exacerbate fertility concerns.^25^ We used qualitative methods to probe complex constructs in a relatively small sample. As such, our study is exploratory in nature but yields insights that may inform future qualitative and quantitative research into young people’s fertility concerns.

Our findings carry implications for clinical counseling and health communication more broadly. Sexual and reproductive health messages aimed at young adults overwhelmingly center on the prevention of pregnancy with little emphasis on factors that promote or impair fertility. Health care providers and educators can play a key role in addressing young people’s fertility questions, concerns, and misconceptions, even for those who are currently hoping to avoid pregnancy. Without such proactive efforts, young people will fill information gaps from other sources that are likely to include the internet and social media. Almost half of U.S. teens report being online almost constantly, and adults aged 18–29 years are more likely than their older counterparts to get news from social media or online searches.^26,27^ Misinformation and sensationalized narratives about sexual and reproductive health abound in these settings.^28,29^ Our findings suggest that successful approaches will be nuanced, increasing awareness and knowledge about fertility without stoking fear. Future research should identify specific strategies that strike this balance and that can be easily integrated into clinical counseling and educational efforts.

## Conclusions

Our study suggests that there are multiple contributors to the phenomenon of perceived infertility among adolescents and young adults, who are statistically at peak fertility. Moreover, young people AFAB with fertility concerns may not necessarily believe they are *currently* infertile but instead harbor anxiety about infertility in the abstract. Sexual and reproductive health messaging for young people should broaden beyond pregnancy prevention to proactively dispel misconceptions and alleviate fears related to fertility and infertility.

## Data Availability

The qualitative dataset analyzed for the current study is not publicly available due to privacy and regulatory considerations. Data are available from the corresponding author upon reasonable request.

## Notes

^a^
Terminology varies across disciplines and between clinical and lay settings. We use *fertility* and *infertility* in recognition of their colloquial use, rather than more technical terms *fecundity* and *subfecundity*.

## Abbreviations used

AFAB: Assigned female at birth
IUD: Intrauterine device
PCOS: Polycystic ovary syndrome
