# Supplementary material for: Perceived Infertility or Fertility Anxiety? Qualitative Insights from Young Adults Attending Reproductive Health Centers
Source: Womens Health Rep (New Rochelle). 2025 Nov 19;6(1):1228–35. doi: 10.1177/26884844251399090 (PMC12677282; doi:10.1177/26884844251399090)
Supplement: Supplementary Data [file 26884844251399090_supplementary_data.docx]

**Supplementary Material: Focus group guide**

*Facilitator: Before we get started, I want to cover a couple of important ground rules about our*

*discussion today. First, there are no right or wrong answers. We are interested in learning about*

*your thoughts and opinions – you are the experts! Second, please keep everything confidential;*

*everything said in the group stays in the group. Thank you for respecting everyone’s privacy.*

*As I mentioned earlier, the discussion is going to be recorded. I’m going to start that right now.*

1. To start out, let’s go around and share one thing that we’re looking forward to this next week.

I’ll start…[facilitator provides response].

2. Now I’d like your thoughts on a couple of terms. “Fertility” – what does that mean to you?

What comes to mind? How about “infertility?”

*Facilitator: For the purposes of this discussion, let’s define fertility as someone’s ability to get*

*pregnant or to get someone pregnant and infertility as the inability to get pregnant or to get*

*someone pregnant.*

3. What are some ways people can tell if they’re fertile or infertile? (Probe: Other than

attempting pregnancy.)

4. What are some things that might cause someone to be infertile?

5. What are some reasons that you, or people you know, worry about their fertility?

6. Now, imagine you wanted a baby right now and started trying. How easy or difficult would it

be for you to get pregnant? Why?

7. Some people think they are infertile or will have trouble getting pregnant even though they’ve

never tried. Why do you think that is? Any reasons you can think of?

8. Think about anyone you know who has struggled to get pregnant or lost a pregnancy. Can

you share how their experience has affected how you think about your own fertility?

9. Some researchers and doctors wonder whether people who worry about their fertility are less

likely to use birth control, even though they don’t want to get pregnant right now. Based on your

own experience or people you know, does this sound right? What do you think?

10. What are your main sources of information about fertility and infertility?

11. What conversations have you had with healthcare providers about fertility or infertility? What

did you like? What did you not like, or was missing? [If none:] What would you think about your

healthcare provider discussing fertility?

12. To wrap up, let’s share any final thoughts about anything we have discussed today.
